# Determination of Carbon Chain Lengths of Fatty Acid Mixtures by Time Domain NMR

**DOI:** 10.1007/s00723-017-0953-2

**Published:** 2017-09-30

**Authors:** E. Nikolskaya, Y. Hiltunen

**Affiliations:** Fiber Laboratory, South-Eastern Finland University of Applied Sciences, Vipusenkatu 10, 57200 Savonlinna, Finland

## Abstract

Average carbon chain length is a key parameter that defines the quality of liquid biofuels. In this paper, a method for the determination of carbon chain lengths of fatty acid mixtures is presented. The approach is based on proton relaxation rates measured by time domain nuclear magnetic resonance. The spin–spin relaxation rates *R*_2_ were used for the estimation of the carbon chain lengths. The method was examined for the set of samples with different mean lengths of the main linear carbon chain. Samples were prepared using four different fatty acids and mixtures of two, three or four of these fatty acids. The correlation coefficient between the known and measured values was equal to 0.994. Based on the relaxation theory, a linear-like dependence between the relaxation rate and carbon chain length was briefly shown, which endorses the experimental results. The developed methodology for determining carbon chain lengths offers robustness and rapidity, which are significant advantages when it comes to online use of the method in real industrial environments.

## Introduction

In recent years, the increasing use of liquid biofuels and the tightening of environmental legislation have created a need for faster and more exact evaluation of fuel properties. Determination of averaged carbon chain length (CL) is important for liquid fuels, because it is one of the key parameters defining their quality and properties. CL also strongly affects other quality parameters, such as viscosity, higher heating value and cetane numbers of liquid fuels [1, 2]. The parameters of liquid fuels are determined based on international standards, which are time consuming, complex and difficult when applicable to online measurements.

High-resolution nuclear magnetic resonance (NMR) is one of the most informative analytical methods in chemistry. ^1^H and ^13^C NMR spectroscopy are widely used in oil and fat analysis [3]. For example, these methods have been applied for the determination of biological origin of vegetable oils [4] and to the quantitation of fatty acids profile of vegetable oils [5, 6]. The techniques based on ^1^H NMR spectroscopy have been intensively developed with the aim of biodiesel composition characterization [7], monitoring the biodiesel production process [8] and evaluation of biodiesel concentration in biodiesel/diesel blends [9–11]. Furthermore, ^1^H NMR spectroscopy has been presented as an alternative to gas chromatography (GC) and near-infrared (NIR) methods for routine analysis of biodiesel and gasoline [11].

However, in spite of high informativeness, accuracy and vast area of applications, high-resolution NMR spectroscopy is, in general, not applicable in industrial environments [12], because of the large size and big weight of the magnet system, the necessity to use a cryogen, the hazard of stray magnetic fields of superconducting magnets [12] and their high cost. On the contrary, small, low field or time domain NMR systems (LF-NMR or TD-NMR) equipped with permanent magnets are available for online instruments in production environments [13]. TD-NMR has become highly attractive for industrial applications due to mobility, relatively low price, easy operating, short time of analysis and a simple and non-destructive sample preparation procedure [12–14]. In particular, the robustness has made TD-NMR a standard analytical tool in quality control. The most known applications of TD-NMR, confirmed by international standards, are solid fat content determination in food and water and oil content in oilseeds [15, 16]. The international standard for hydrogen content determination in aviation fuels [17] has also been developed.

Liquid fuel analysis by TD-NMR is continuously growing. For example, the application of 1D *T*_2_ relaxation times distribution and 2D cross-correlation *T*_1_−*T*_2_ analysis to the characterization of potential biodiesel resources are highlighted in the paper [18]. The possibility of ex situ monitoring of the transesterification reaction by unilateral NMR is shown in the work by Cabeça et al. [19]. In most works related to TD-NMR applications to liquid fuels, the main findings are based on the correlation between *T*_2_ relaxation time and viscosity [20, 21] and also other different fuel parameters—total acid number, refractive index, API gravity [20], cetane number and iodine value [21]. Experimentally obtained correlations of *T*_2_ on viscosity and other physical parameters for different kinds of liquid fuel samples, oils, alkanes [20–23] were described empirically in most of the investigations. An attempt to give an interpretation of *T*_2_ dependence on viscosity for alkanes was given in [22] using the NMR relaxation theory, considered for the shape of spherical molecules.

In this work, we have applied TD-NMR to study molecular structures of fatty acids. The correlation between relaxation rates and average carbon chain lengths of fatty acid mixtures showed the possibility of TD-NMR as a method for rapid measurement of the average CL of liquid fuels.

## Materials and Methods

### Sample Preparation

Samples with different average carbon chain lengths were prepared as different mixtures of several common fatty acids (Table 1). The fatty acids for this study were selected by their melting points, which should be lower than 25 °C to perform the measurements at room temperature. The commercially available fatty acids were used without further purification. Their properties (molecular weights, melting points and carbon chain lengths) are given in Table 1.

**Table 1:** Fatty acids used for samples preparation

Common (IUPAC) name	Chemical formula	Molecular weight, g/mol	Melting point, °C	Carbon chain length CL	Producer
Butyric (butanoic) acid	C_4_H_8_O_2_	88.11	−5.1	4	SIGMA-Aldrich
Caproic (hexanoic) acid	C_6_H_12_O_2_	116.16	−3.4	6	SIGMA-Aldrich
Caprylic (octanoic) acid	C_8_H_16_O_2_	144.21	16.7	8	SIGMA-Aldrich
Oleic (9*z*-octadec-9-enoic) acid	C_18_H_34_O_2_	282.46	13.0	18	MERCK

Average carbon chain length CL_mix_ values of the samples can be calculated knowing the molecular weights and mass parts of fatty acids using the following equations:

1 $${\text{CL}}_{\text{mix}} = \mathop \sum \limits_{i = 1}^{n} a_{i} {\text{CL}}_{i } ,$$

where *a*_*i*_ is the part of molecules of *i*-acid in mixture, *n* is the number of acids in mixture and CL_*i*_ is the carbon chain length of *i*-acid. The *a*_*i*_ is obtained from the equation:

2 $$a_{i} = \frac{{N_{i} }}{{\mathop \sum \nolimits_{i = 1}^{n} N_{i} }} ,$$

where the number of molecules of *i*-acid, *N*_*i*_, is calculated from Eq. (3):

3 $$N_{i} = \frac{{m_{i} N_{A} }}{{M_{{{\text{w}}i}} }} ,$$

where *m*_*i*_ is the mass part of *i*-acid in the mixture (in g), *M*_w*i*_ is the molecular weight of *i*-acid (in g/mol) and *N*_*A*_ is the Avogadro constant.

Twenty samples were selected for the current study. The samples had the CL_mix_ values within the range from 4 to 18. These values are found in Table 2.

**Table 2:** Experimental values of carbon chain lengths CL_mix_ and spin–spin relaxation rates *R*_2_ of fatty acid mixtures

Sample	Mass fraction				CL_mix_	*T*_2_, s	*R*_2_, s^-1^
	CL						
	4	6	8	18			
1	0.25	0.25	0.25	0.25	7.11	0.53	1.90
2	0.3	0.3	0.4		5.86	0.66	1.52
3		0.35	0.5	0.15	7.90	0.48	2.08
4		0.2	0.5	0.3	9.15	0.41	2.43
5		0.5	0.4	0.1	7.32	0.48	2.07
6		0.15	0.15	0.7	12.62	0.25	3.98
7	1				4.00	0.93	1.07
8		1			6.00	0.66	1.50
9			1		8.00	0.47	2.13
10				1	18.00	0.17	5.79
11	0.7		0.3		4.83	0.75	1.33
12	0.5		0.5		5.52	0.67	1.49
13	0.3		0.7		6.35	0.58	1.72
14			0.7	0.3	9.80	0.35	2.88
15			0.5	0.5	11.38	0.29	3.49
16			0.3	0.7	13.44	0.25	4.06
17		0.2		0.8	13.46	0.26	3.89
18		0.5		0.5	9.50	0.40	2.51
19		0.8		0.2	7.12	0.54	1.84
20			0.2	0.8	14.71	0.22	4.60
21			0.6	0.4	10.54	0.33	3.01
22			0.8	0.2	9.13	0.39	2.56

### TD-NMR Measurements

The TD-NMR measurements were done using the portable NMR analyser Spin Track [24] with ^1^H resonance frequency of 25.7 MHz. The diameter of the sensor hole was 10 mm. The digital receiver console provided quadrature acquisition with the ringing time of around 10–12 μs. CPMG (Carr–Parcell–Meiboom–Gill) pulse sequence [25] was applied for the measurement of spin–spin relaxation rate *R*_2_. Echo time was equal to 6 ms; the number of 180° pulses in the sequence was 600. Relaxation delay was 2 s, and the amount of scans was equal to 8. Durations of 90° and 180° RF pulses were 7 and 14 μs, respectively. All measurements were performed at room temperature (25 °C).

The experimental relaxation rate *R*_2_ is dependent on the composition of different fatty acid molecules in a sample [26]:

4 $$R_{2} \; = \;\mathop \sum \limits_{i = 1}^{n} \left( {P_{i} R_{2i} } \right),$$

where *P*_*i*_ and *R*_2*i*_ are the proton populations and spin–spin relaxation rates of *i*-th fatty acid molecule; *n* is the number of fatty acid types in mixture.

**Fig. 1:**
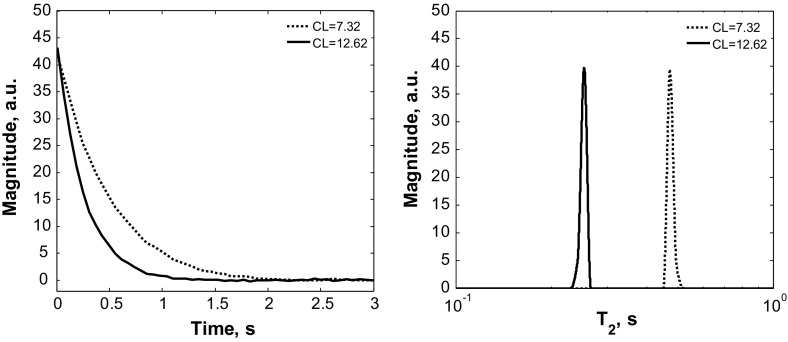
Laplace transformation applied to two measured CPMG decays

The individual *R*_2*i*_ values corresponding to different fatty acid molecules in a mixture were not observed due to fast proton exchange [26]. It takes place as the magnetization exchange rate *k*_ex_ between fatty acid molecules is much higher than the measured relaxation rate. Thus, one *R*_2_ distinct value was obtained for each sample. It was confirmed by applying Laplace transformation to measured CPMG decays, as can be seen in Fig. 1. Therefore, all measured CPMG decays *A*(*t*) were fitted by mono-exponential function defined by Eq. (5)

5 $$A\left( t \right) = A_{0} {\text{e}}^{{ - R_{2} t}} ,$$

where $A_{0}$ is the maximal signal magnitude and *R*_2_ the spin–spin relaxation rate. A Matlab software script, written by the authors, was used for determining *R*_2_ values.

### Theory

The Bloembergen–Purcell–Pound (BPP) approach [27] is normally used for the description of spin–spin $T_{2}$ NMR relaxation in liquids. According to BPP theory [25, 27], spin–spin relaxation time $T_{2}$ or relaxation rate $R_{2}$ ($R_{2} = 1/T_{2}$) is dependent on molecular mobility expressed as correlation time $\tau_{\text{c}}$, which is the characteristic parameter of molecular mobility. For liquid samples $\omega_{0} \tau_{\text{c}}$ ≪1 ($\omega_{0} =$ the resonance frequency of TD-NMR devices) and *R*_2_ linearly increases with the growth of correlation time as follows:

6 $$R_{2} = 10M_{2} \tau_{\text{c}} ,$$

where $M_{2}$ is the value of second moment, which is determined by the strength of dipole–dipole interactions between neighbouring nuclei.

The correlation time in BPP equations can be described by Stokes–Einstein–Debye equation [28, 29]:

7 $$\tau_{\text{c}} \; = \;\frac{{C_{\text{r}} \eta V}}{{k_{\text{B}} T}},$$

where $V$ is the molecule’s effective volume [30], $\eta$ is viscosity, *T* is temperature, $k_{\text{B}}$ is Boltzmann constant and $C_{\text{r}}$ is fitting parameter determined by experiment [28–31]. The Stokes–Einstein–Debye equation is often used in the form modified for homogeneous fluids with molecules described as spheres with hydrodynamic or Stokes radius, not molecular.

Rotational movements are the most perceptible to NMR relaxation contribution, whereas the frequency range of translational motions makes a little contribution to the NMR relaxation proton spectra, and vibrations due to their high frequency are not observed by NMR relaxation.

Correlation times for different molecular motions are the time parameters in specific correlation functions. Complex modelling is required to describe them separately. On the other hand, rotational correlation times of molecules can be calculated by known corresponding diffusion coefficients, which are described by the Stokes–Einstein equation [29]:

8 $$D_{\text{r}} = \frac{{k_{\text{B}} T}}{{8\pi \eta R^{3} }},$$

where $D_{\text{r}}$ is rotational diffusion coefficient, $k_{\text{B}}$ is Boltzmann constant, *T* is temperature, $\eta$ is viscosity and *R* is hydrodynamic (Stokes) radius of molecule.

When describing the rotational movement of molecules, it is convenient to consider that molecules have a spherical shape with hydrodynamic radius *R*. In the consideration that the molecule’s effective volume is equal to

9 $$V = \frac{{4\pi R^{3} }}{3},$$

the combination of the Eqs. (7–9) gives the following result [28]:

10 $$\tau_{\text{c}} \sim \frac{1}{{D_{\text{r}} }}.$$

Thus, it is obviously seen that the rotational correlation time is dependent on the corresponding diffusion coefficient (Eq. 10). Equation (10) is the widely used ratio for calculation of the NMR relaxation correlation time [22], as rotational molecular movements are the major contributors to NMR relaxation.

Generally molecules of different shapes can be characterised by hydrodynamic radii, thus Eq. (10) is probably applicable both to unbranched, rod-like molecules, which are mostly represented in liquid biofuels and to different types of paraffins and aromatic hydrocarbons, which are presented in petroleum fuels.

To connect the rotational diffusion coefficient with the molecular weight, the well-known ratio for self-diffusion *D*_self_ behaviour description for untangled polymer chains [32, 33] can be used. In case of elongated, unbranched molecules, there is direct dependence between these two parameters:

11 $$D_{\text{self}} \sim M_{\text{w}}^{ - \alpha } \;\;{\text{for}}\;M_{\text{w}} < M_{\text{wc}} ,$$

where *M*_wc_ is the entanglement coupling molecular weight [32], *α* is a coefficient, equal to 1.

Combining the expressions (6), (10) and (11), it is clearly seen the linear relationship between *τ*_c_ and, therefore, of *R*_2_ with molecular weight *M*_w_:

12 $$R_{2} \sim M_{\text{w}}$$

It is easy to suppose that in the case of unbranched hydrocarbons with similar chemical organisation the molecular weight is proportional to molecular size, and the latter one can be evaluated by CL. Relying on the dependence of *R*_2_ on molecular weight *M*_w_ (Eq. 12), it is assumed the linear-like dependence of *R*_2_ on CL:

13 $$R_{2} \sim {\text{CL}} .$$

## Results and Discussion

The CPMG decays for all fatty acid samples were measured and then fitted by mono-exponential function for determining spin–spin relaxation rates *R*_2_. The results of *R*_2_ values are collected in Table 2. The average carbon chain lengths CL_mix_ are plotted as a function of the relaxation rates *R*_2_ in Fig. 1 showing a linear relationship. By applying the linear fitting to the data set, the model can be calculated, as follows:

14 $${\text{CL}}_{\text{NMR}} = a\ {R_{2}} + b,$$

where *a* = 2.914 s ± 0.069 s and *b* = 1.507 ± 0.198.

**Fig. 2:**
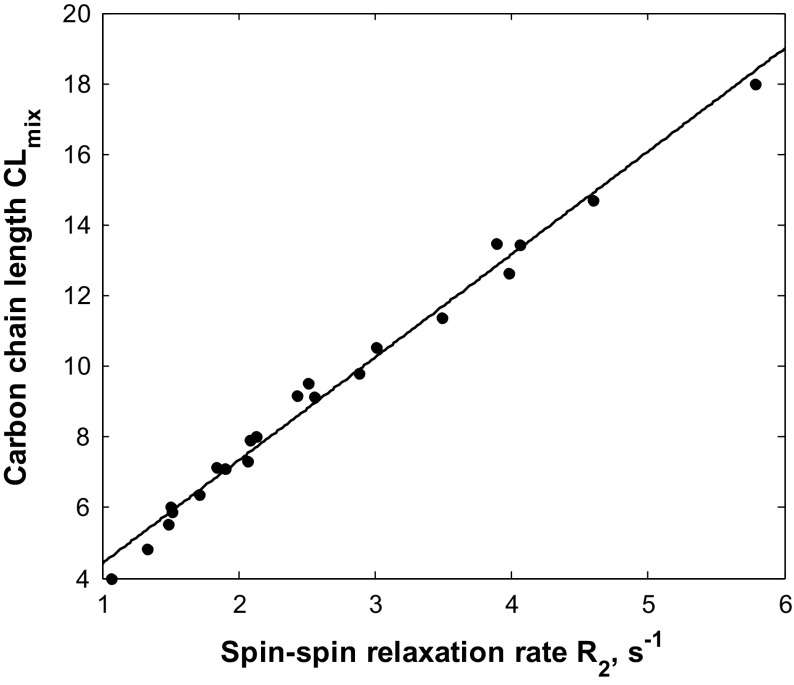
Average carbon chain lengths CL_mix_ are plotted as a function of the relaxation rates *R*_2_. The solid line is fitted by a linear model

The values defined by Eq. (14) are in a good agreement with the CL_mix_ values, as shown in Fig. 2. The correlation coefficient between these two data sets is equal to *R* = 0.994 and standard error to 0.384. The errors can be caused by errors in *R*_2_ determination (fitting errors) and sample preparation procedure (errors of weighting of fatty acid mass portions and mixing).

In the present study, carbon chain lengths of fatty acid mixtures have been determined by the time domain NMR, showing a good agreement with the known ones. The fatty acids were chosen by their melting points, so that it was lower than the room temperature. However, considering the method for biofuel production quality control, it should be taken into account that the fatty acid profile in real vegetable oils includes typically saturated palmitic acid (C16:0), stearic acid (C18:0), unsaturated oleic (C18:1) and linoleic acid (C18:2), [18, 34]. The specific profiles can also contain saturated and unsaturated acids of higher carbon chain lengths (more than 20) [35] besides the main components. So the carbon chain lengths of biofuels are typically longer than CLs used in the present study. As can be seen from Eq. (13) and our experimental results presented here, the relaxation rates of fatty acid molecules depend linearly on carbon chain lengths. It is quite clear that this relationship is valid in the case of longer molecules and thus also for biofuels.

In the view of better quality of biodiesel, relatively long-chain saturated and exactly monounsaturated acids are the most important because this combination will provide higher oxidation stability; the presence of double bond will decrease the melting temperature, viscosity, cloud point and pour point; and the long CL will guarantee a high enough cetane number and heat of combustion [1]. The effect of the different amount of double bonds to the NMR relaxation was not investigated in the current work. However, as the presence of double bond increases the mobility (correlation time) of protons, as a result, some effect on relaxation rate *R*_2_ is probably expected to be observed.

In this paper, a method for the determination of carbon chain lengths of fatty acid mixtures is presented. The approach is based on proton relaxation rates measured by TD-NMR. In our earlier paper [36], we have demonstrated the possibilities of TD-NMR in online analytics. The method for determining carbon chain lengths offers robustness and rapidity, which are significant advantages when it comes to online use in real industrial environments.

## Conclusion

The results presented here illustrate that NMR relaxation can be used for the determination of average carbon chain lengths, which is one of the key parameters, for example, defining the quality properties of biofuels. The results also demonstrate the possibility of TD-NMR applying to industrial process control of liquid biofuels, and therefore, monitor their product quality, which will be still more important for the tightening environmental legislation.
